# A Micro-Computed Tomography Comparison of the Porosity in Additively Fabricated CuCr1 Alloy Parts Using Virgin and Surface-Modified Powders

**DOI:** 10.3390/ma14081995

**Published:** 2021-04-16

**Authors:** Mirko Sinico, Suraj Dinkar Jadhav, Ann Witvrouw, Kim Vanmeensel, Wim Dewulf

**Affiliations:** 1 Department of Mechanical Engineering, KU Leuven, Celestijnenlaan 300 Box 2420, 3001 Leuven, Belgium; ann.witvrouw@kuleuven.be; 2 Member of Flanders Make—Core Lab MaPS, KU Leuven, 3001 Leuven, Belgium; 3 Department of Materials Engineering, KU Leuven, Kasteelpark Arenberg 44 Box 2450, 3001 Leuven, Belgium; suraj.jadhav@kuleuven.be (S.D.J.); kim.vanmeensel@kuleuven.be (K.V.)

**Keywords:** computed tomography, porosity analysis, surface-modified CuCr1 powder, laser powder-bed fusion, additive manufacturing, laser reflectivity

## Abstract

Recently, the use of novel CuCr1 surface-modified powder for reliable laser powder-bed fusion (LPBF) manufacturing has been proposed, enabling a broader LPBF processing window and longer powder storage life. Nevertheless, virgin CuCr1 powder is also LPBF processable, on the condition that a high-energy density is employed. In this work, we compare two dense specimens produced from virgin and surface-modified CuCr1 powder. Furthermore, a third sample fabricated from surface-modified powder is characterized to understand an abnormal porosity content initially detected through Archimedes testing. Utilizing high-resolution micro-CT scans, the nature of the defects present in the different samples is revealed. Pores are analyzed in terms of size, morphology and spatial distribution. The micro-CT data reveal that the virgin CuCr1 dense specimen displays keyhole pores plus pit cavities spanning multiple layer thicknesses. On the other hand, the sample fabricated with the surface-modified CuCr1 powder mainly contains small and spherical equi-distributed metallurgical defects. Finally, the CT analysis of the third specimen reveals the presence of a W contamination, favoring lack-of-fusion pores between subsequent LPBF layers. The LPBF melting mode (keyhole or conductive), the properties of the material, and the potential presence of contaminants are connected to the different porosity types and discussed.

## 1. Introduction

Porosity is an important subject in the additive manufacturing (AM) research field. Desired or engineered porosity is utilized nowadays for the production of bio-compatible metal implants and prostheses [1,2], bio-active ceramic scaffolds [3,4], geopolymer filters for water treatments [5], breathable steel for molding components [6], and multiple other applications [7]. The intrinsic design freedom of the additive process grants that the porosity can be engineered to a level of detail that is governed by the accuracy and precision of the process itself [8]. However, desirable porosity is only the positive side of the story, and most often, pores are seen as defects hampering the successful obtainment of fully dense AM components.

Undesirable porosity has been the matter of numerous studies for AM polymers, metals and ceramics alike [9,10,11], specifically for the materials presenting unfavorable properties which thwart some AM fabrication routes. In powder-based metal AM research, a good example of this adverse conjunction of material properties and process conditions has been the fabrication of copper parts through the laser powder-bed fusion (LPBF) process, also known as selective laser melting (SLM). During LPBF fabrication, a laser source typically in the near-infrared range shines over deposited layers of fine metal powders to create the object slice-by-slice. While the available material palette for LPBF is continuously increasing [12], some materials like gold, silver, and copper are not easily processable because of their high thermal conductivity in conjunction with their low absorptivity for the near-infrared laser radiation [13]. Hence, either different production routes had to be employed or a high level of porosity, typically ‘lack-of-fusion’ defects between consecutive layers, was to be accepted for the fabricated parts. Thanks to new advancements, the processability problem of pure copper or high-copper-containing alloys has been almost completely solved. It concerns both LPBF machine hardware improvements, with lasers having higher power or different emitted wavelengths [14,15], and advancements on the proper selection and modification of the starting raw powder material, with surface-modified powders [16,17,18] or tuned copper alloys [19,20]. Nevertheless, still a higher porosity and higher number of defects are expected from parts manufactured in pure or nearly pure copper, with densities often below the industrial average requirement of ≥99.7% for LPBF. For example, densities up to 99.1% were reported by Yan et al. for pure copper; similar densities were achieved by Jadhav et al. [21] and others [22] with the employment of a high-power (≥1 kW) fiber laser. In the realm of high-copper-content alloys, densities above >99% are reached for, e.g., CuCr1Zr (99.84% in [19]), CuSn0.3 (99.6% in [17]), or CuCr1 (99.1% in [16]).

The production of functional copper parts is not impeded if the part density is only slightly below the 99.7% industrial standard [16,22,23]. However, the remaining porosities should be equally distributed inside the object and preferentially spherically shaped to avoid having an anisotropy of the mechanical and thermal/electrical properties or critical points of failure due to local stress concentration upon mechanical loading. Determining the porosity information is challenging, because the common Archimedes or metallographic inspections are not able to derive the 3D shape and spatial distribution of the pores inside a sample. Nevertheless, parts additively fabricated with the same material and achieving the same relative density can demonstrate very different mechanical and thermal performances depending on how the pores are positioned and shaped [9,24]. This is again more critical for copper, because normally its optimal LPBF processing parameter window is very narrow; hence, small variations in laser power or scan speed, for example, can result in a sudden change of the melt pool dimensions and melting behavior [25]. Accordingly, defects like lack of fusion, keyholes, or cavities [9] can be formed when only small spherical pores (e.g., metallurgical or from stochastic gas entrapment) would be acceptable.

A better comprehension of the porosity development inside AM parts can be obtained using high-resolution computed tomography (CT) instruments [26]. X-ray micro-CT has the advantage of recreating a full 3D model of a scanned object, thus allowing the non-destructive inspection of internal features or defects. For this reason, micro-CT is being used more and more as a method to dimensionally characterize both desired, structured porosity and undesired defects of AM objects, and the advancements in this research field are adequately summarized in recent reviews [27,28,29]. On the other hand, it is widely accepted that CT is far from being a perfect inspection method, with multiple factors influencing the final CT scan quality. Specifically, for porosities, the limited achievable CT spatial resolution and voxel resolution of common micro-CT systems impedes on average the detection of the smaller defects, like the metallurgical or gas porosities, in metal AM parts even with optimized CT conditions. The measured size of the pores can moreover be impacted from voxel size and segmentation errors [30]. Nonetheless, CT remains the only available technique that is able to non-destructively inspect the geometry of internal structures.

CT investigations of defects in LPBF copper are sparsely available [31,32]. Challenges encountered when scanning Cu specimens are attributed to the high X-ray mass attenuation coefficient of copper [33]. This forces the use of higher kV and μA for the CT scan, leading to a loss in spatial resolution due to the increase in the X-ray gun focal spot size [34]. Hence, high care must be taken on the selection of both CT scan parameters and specimen size, depending on the specifications of the available CT machine and the desired resolution to be achieved.

In this work, high-density copper specimens produced out of an innovative surface-modified CuCr1 powder are compared, through micro-CT investigations, against an analogous high-density sample fabricated with a virgin CuCr1 powder. The specimens have been selected out of the samples used in the research described in [16], where an innovative powder surface-modification method has been found to enhance the optical absorption of CuCr1 copper powders for the LPBF process. The formation of a thin rim of metallic chromium and chromium nitrides on the outer surface of the powders was reported to considerably increase the optical absorption of the near-infrared fiber laser light commonly used in LPBF machines and, furthermore, to increase the powder storage life due a lower tendency toward oxygen pickup. The novel surface-modified CuCr1 powder was compared to the virgin CuCr1 powder in [16] in terms of LPBF processability and was characterized for final mechanical, electrical, and thermal properties of built parts. An in-depth analysis of the resulting porosity statistics was however not included, as it was not the focus of the research. Relative porosity content of the LPBF produced specimens was instead evaluated by Archimedes testing and from standard metallographic sections.

The goal of this follow-up research is to further cast light on the amount, morphology and spatial distribution of the porosities for the different CuCr1 specimens, exploiting the information of high-resolution micro-CT scans. In this manner, we want to determine how the pore characteristics are changing, depending on the different starting LPBF processing conditions and to unravel, if relevant, potential property anisotropy. In addition, the CT evaluation of an extra contaminated CuCr1 specimen is described to show how CT can help the optimization of the new LPBF material.

## 2. Materials and Methods

### 2.1. LPBF Specimens Production and Initial Characterization

Three copper LPBF samples are the subject of this work. Two of them, denominated virgin CuCr1 (V-CuCr1) and surface-modified CuCr1 (N2-CuCr1), have been selected directly out of the samples used in the research described in [16].

**Figure 1:**
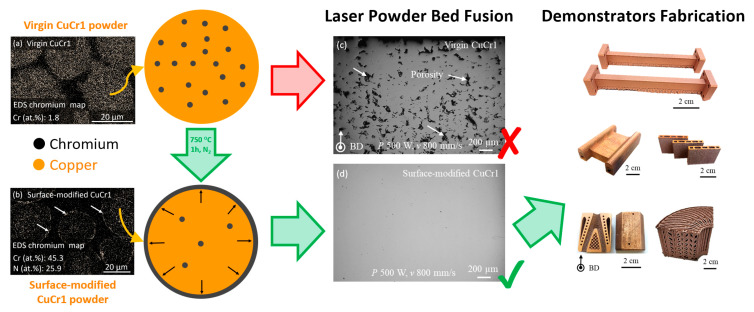
Graphical summary of the research of Jadhav et al. [16] concerning the development of surface-modified CuCr1 powder for reliable laser-based additive manufacturing; insets (**a**,**b**) present the energy-dispersive X-ray spectroscopy (EDS) chromium map of the cross-section of the virgin and of the surface-modified CuCr1 powder particles, respectively; insets (**c**,**d**) are cross-sectional surfaces of LPBF samples from virgin and surface-modified powders respectively (with BD indicating the building direction). Modified with permission from Ref. [16], 2020 Elsevier.

Starting from pre-alloyed, argon atomized CuCr1 powder with D50 of 38.8 μm, the pre-processing of the powder for its surface modification was optimized by Jadhav et al. [16] to achieve an outward diffusion of chromium toward the particles surface, favored by the presence of a surrounding nitrogen atmosphere at a temperature of 750 °C (1 h treatment-time). In such a way, the surface-modified layer gets enriched in metallic chromium and chromium-nitrides. This external rim, of around 459 ± 50 nm, was the key discovery of the research discussed in [16] to obtain a reliable and efficient CuCr1 LPBF processing, where only 20% of the volumetric energy density (Ev) is required to obtain dense parts in comparison to the processing of virgin CuCr1 powder. This beneficial effect is a consequence of the enhanced (68% at 1080 nm) optical absorption, of the surface-modified formulation, for the near-infrared laser radiation. The feasibility of the LPBF processing for the surface-modified CuCr1 powder was tested not only on academic specimens but as well as on industrial demonstrators with various dimensions and complexity. Moreover, the surface-modified layer was proved to be a fugitive layer [16,35], given the fact that the nitrogen is released during the laser melting and therefore does not alter the chemical composition of the parts nor induces the formation of chromium-nitrides precipitates. Figure 1 presents a graphical summary of the surface modification, its impact on LPBF CuCr1 part density, and examples of the fabricated demonstrators.

In like manner, for this work, samples V-CuCr1 and N2-CuCr1 were fabricated in an in-house-developed LPBF machine equipped with a continuous, single-mode, 1 kW fiber laser, emitting light with a wavelength of 1080 nm, and a beam diameter of 40 μm. The CuCr1 specimen made with the virgin powder batch was manufactured with a LPBF parameter set having 200 mm/s of laser scan speed (*v*), 500 W of laser power (*P*), and a corresponding *E_v_* of 926 J/mm^3^. The specimen made of surface-modified CuCr1 powder was produced with a laser scan speed of 800 mm/s, 500 W of laser power, and a corresponding energy density of 231 J/mm^3^. Hatch spacing (*h*) of 0.09 mm and a layer thickness (*t*) of 0.03 mm were constant for both samples. The scanning strategy employed was a zigzag (bi-directional) scan strategy within each layer, with a 90° scan rotation angle between the successive layers. Further details on the production route and the general properties of utilized powders and final parts are described in [16].

A last third specimen in examine was a N2-CuCr1 sample made, unintentionally, from a tungsten-contaminated surface-modified CuCr1 powder batch (sample labelled as CN2-CuCr1). During the multiple LPBF build jobs with the innovative powder, a batch of samples was identified having an unexpected high porosity content, even though it was manufactured using the optimum set of parameters listed before for N2-CuCr1. Micro-computed tomography investigation was therefore employed to understand the source of the porosity.

Before the CT analysis, a recap of the Archimedes density behavior of LPBF parts from the two CuCr1 formulations is presented at the beginning of Section 3, together with exemplificative polished and etched cross-sections of the three samples, to qualitatively compare the data against the CT results. For the etched sections, a standard Keller’s reagent made of 3.5 g FeCl_3_, 2.5 mL HCl, and 75 mL C_2_H_5_OH was employed.

### 2.2. Micro-Computed Tomography Analysis

The samples described in Section 2.1 were subjected to a micro-CT evaluation utilizing a Nikon XTH 225ST (Nikon Metrology Europe NV, 3001 Leuven, Belgium) for the investigation of their porosity/inclusion distributions and statistics. Prior to the CT scan, out of each cubic sample, a slice of 8 × 8 × 2 mm^3^ was mechanically cut to reduce the specimen volume, allowing therefore both a higher magnification and spatial resolution for the CT scan itself. Hence, a magnification of 30× was attained with the sample placed as close as possible to the X-ray’s source. This magnification allowed the achievement of a voxel size of 6.7 μm for the 3D datasets; the other CT acquisition settings such as the voltage, the current, and the exposure time were set, respectively, at 195 kV, 41 μA, and 4000 ms. The high exposure time was necessary to compensate both for the low scanning power, 8 W, and the installed copper filter of 1 mm. The copper filter applied in front of the X-rays source was deemed advantageous to harden the X-rays beam before reaching the specimen itself, partially reducing the beam hardening artefact. On the other hand, the low scanning power was chosen to increase spatial resolution by keeping the X-rays source spot size at a minimum level, which as per manufacturer datasheet should correspond to a 4 μm spot for the listed settings [36].

After the scan, each set of 3142 projections was reconstructed with the CT Pro 3D software (v4.4.4, Nikon Metrology Europe NV, 3001 Leuven, Belgium) and loaded on VGSTUDIO MAX (v3.3, Volume Graphics GmbH, 69115 Heidelberg, Germany) for the porosity/inclusion analysis. The surface determination of the acquired samples was performed with the VGSTUDIO advanced option active, employing an iterative search of the surface edge over a search distance of 12 voxels with the starting contour being the ISO50 value. Following, the specimens were aligned in the software Cartesian coordinate system using a plane-line-point alignment, mainly exploiting the 8 × 8 mm^2^ top surface plane plus reference scan tracks in relief on this surface. This initial alignment was important for extracting the porosity information relative to the LPBF building direction (Z axis) and the hatching scan vectors, i.e., perpendicular and parallel to the laser line tracks (X, Y axes). Subsequently, a porosity/inclusion evaluation was performed for each specimen, using the VGSTUDIO-only threshold algorithm. A region of interest of 6.5 × 6.5 × 1 mm^3^ was used instead of the full volume of the samples, to avoid edge effects and to limit the influence of the beam hardening artefact still partially present near the surface. For the porosity identification, the grey value determining the edge between material and void was set equal to the determined surface, and its threshold was calculated in interpolation mode. Porosities were then displayed, for qualitative analysis, in voxel accuracy, and color-coded according to their measured circumscribed sphere diameter (*Ø_cs_*). Inclusions identification was set instead in deviation mode to identify grey values strongly deviating from the main material peak. The smallest reliable pore or inclusion to be detected was limited by 8 times the CT scan voxel size, corresponding to a perfectly spherical particle of around 16 μm in diameter. Finally, all the statistics related to the pores and inclusions were exported in a csv file and further analyzed via an in-house MATLAB code to quantify their size, shape, and spatial distributions.

## 3. Results and Discussion

### 3.1. LPBF Production and Preliminary Characterization

**Figure 2:**
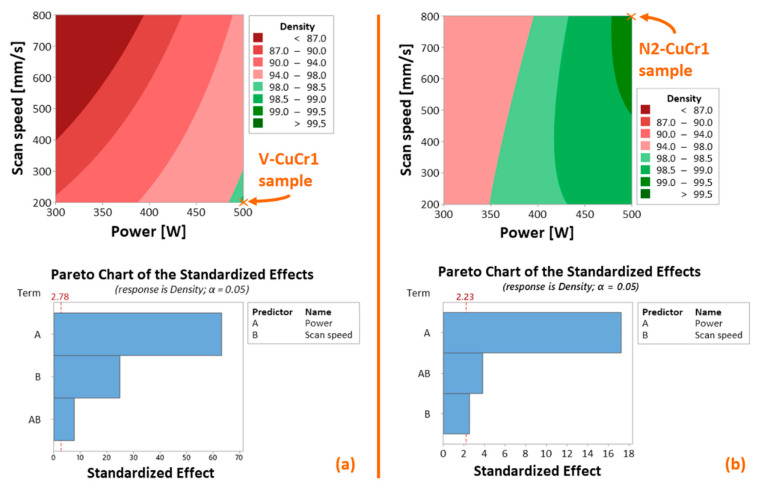
Contour plots of the calculated linear regression models for the LPBF production design of experiment of (**a**) Virgin CuCr1 and (**b**) Surface-modified CuCr1, with corresponding Pareto charts of standardized effects; predictors are laser power [W] and scan speed [mm/s]; response is the measured Archimedes relative density [%]; order two predictors are not displayed since they result as statistically non-significant.

The LPBF manufacturing step was successfully accomplished. To give context to the high-density virgin CuCr1 (V-CuCr1) and surface-modified CuCr1 (N2-CuCr1) specimens produced for this research, the design of experiment (DoE) presented in Figure 7 of [16] has been analyzed with a fitted general linear regression model. The regression model takes into consideration the continuous predictors laser power and scan speed through order two, and their interaction. The continuous predictors are also standardized to mitigate multi-collinearity (keep VIF—variance inflation factors—low). The response factor corresponds to the measured Archimedes relative density. Obtained R^2^ are 99.95% and 99.40% for the virgin CuCr1 DoE (Figure 2a) and the surface-modified CuCr1 DoE (Figure 2b), respectively. On each contour plot, the samples taken in consideration for this work are marked.

The contour plots of Figure 2 clearly show how the novel surface-modified CuCr1 alloy powder grants the attainment of dense LPBF parts within a broader window of laser scan settings. Moreover, the Pareto charts highlight the lower sensitivity of the surface-modified CuCr1 powder to changes in the laser scan speed (predictor B) in comparison to the virgin CuCr1 powder for the range of scan settings taken in consideration. This different dependency might be critical for the laser melting mode experienced during LPBF fabrication (keyhole or conduction mode [37]) and hence can directly affect the properties of the produced copper part even if similar porosity content is reached.

**Figure 3:**
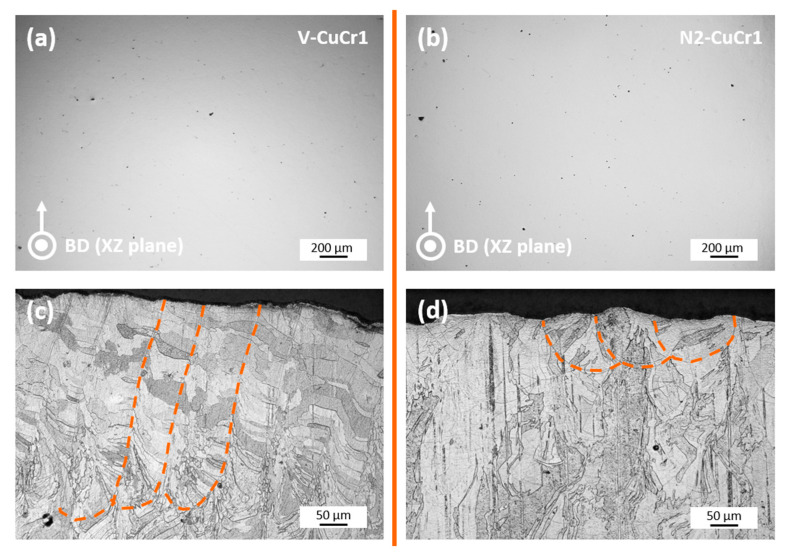
Build direction (XZ plane) cross-sectional surfaces for the (**a**) V-CuCr1 and (**b**) N2-CuCr1 specimens; (**c**,**d**) are etched sections of V-CuCr1 and N2-CuCr1, respectively, which include the top layers of the samples to highlight the general melt pool shape (orange dashed curves).

The marked samples V-CuCr1 and N2-CuCr1 in Figure 2, corresponding to the samples exhibiting the highest relative densities for the modified and virgin (unmodified) case, were the samples selected for the in-depth micro-CT evaluation. However, to qualitatively compare the results of the micro-CT with the initial understanding of the porosity content and LPBF melting mode, both samples were also evaluated using optical microscopy performed on metallographic unetched and etched cross sections. Accordingly, Figure 3 comprises both a metallographic cross-section of the two specimens in the XZ plane (Z is the LPBF building direction) and etched sections, which include the top layers of the samples to highlight the melt pool shapes of V-CuCr1 and N2-CuCr1.

The cross-sectional surfaces reveal expected behaviors of the two specimens. Both unetched polished cross-sections (Figure 3a,b) reflect the high densities achieved, with only minor scattered residual pores visible for the two samples. The measured Archimedes density corresponds to 98.64% and 99.10% for the V-CuCr1 and N2-CuCr1 specimens, respectively. The small difference in the total amount of porosity cannot be easily appreciated on the single unetched cross-sections, also due to the fact that the copper material, being soft and ductile, is very challenging to polish, and consequently, the very small pores can partially become hidden after plastic deformation induced by the metallographic preparation. Moreover, from the single cross-sections, it is difficult to establish a clear trend over the type and 3D spatial distribution of the pores.

In regard to the etched cross sections, Figure 3c,d, an expected different LPBF melting mode between the two specimens is evidenced. V-CuCr1 presents deep melt pools with an average depth greater than 10 times the employed layer thickness, indicating keyhole melting mode. This is compatible with the employed LPBF parameters for the V-CuCr1. Due to the low optical absorption of the virgin copper powder, these parameters were set at a very low laser scanning speed to achieve high density (Figure 2). On the other hand, the N2-CuCr1 sample has semi-elliptical-shaped melt pools with a depth on the order of 2–3 times *t*, indicating a conduction-controlled melting.

**Figure 4:**
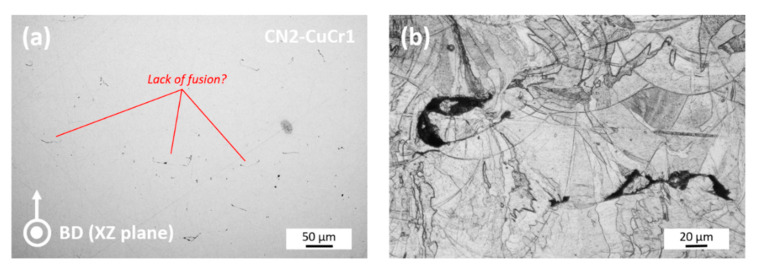
(**a**) Build direction (XZ plane) cross-sectional surfaces for the CN2-CuCr1 specimen; (**b**) zoomed-in etched section to highlight the observed lack-of-fusion porosities.

Lastly, the CN2-CuCr1 sample was characterized. CN2-CuCr1 should theoretically be identical to N2-CuCr1, as the fabrication was performed at the same processing conditions and LPBF parameters but in different build jobs. Per contra, the Archimedes density evaluation of CN2-CuCr1 revealed a porosity content of 1.77%. While a statistical variance of the resulting porosity is indeed possible, an almost 1% difference is normally not expected. Figure 4 presents both unetched and etched cross-sections for the CN2-CuCr1 sample.

An initial inspection of the unetched polished XZ section of the CN2-CuCr1 sample revealed the noticeable presence of knit weld lines, suggesting lack of fusion between consequent LPBF layers, in contrast to the N2-CuCr1 sample. This is corroborated by the etched section (example in Figure 4b), where the lack-of-fusion porosities appear more clearly visible at the bottom boundaries of some melt pools. However, no direct evidence is gathered on the reason behind the unexpected behavior of the CN2-CuCr1 sample. Thereupon, to provide an answer on the porosity causes and distributions of the three selected CuCr1 specimens, the results of the micro-CT evaluation are presented in the next section.

### 3.2. Micro-Computed Tomography Results

The micro-CT evaluation is presented, with an in-depth discussion on the porosity and inclusion analyses for each of the three CuCr1 samples, and the possible connection between defect characteristics and the expected material/part properties. The labeling style for the different types of pores follows the review of Sola and Nouri [9]. The small porosities (up to tens of μm) are hereafter defined as metallurgical pores, referring to all the small defects not directly relatable to a specific source of porosity within the current investigation. Hence, small defects coming, e.g., from pores entrapped in the original feedstock powder or pores from powder compaction stochastic fluctuations, being not discernible, also fall in the category of metallurgical pores.

It must be noted that Archimedes porosity count and CT porosity count cannot be directly compared, as the resolution and accuracy of the latter are limited by both the CT scan settings and the employed thresholding method. Studies on the factors influencing the porosity determination accuracy of both CT and Archimedes methods have been reported in different publications [38,39,40]. However, it is possible for the samples in examine to directly compare the CT results among each other, since specimens of same size and overall composition were scanned and analyzed with identical CT parameters.

#### 3.2.1. Micro-CT of the V-CuCr1 Specimen

**Figure 5:**
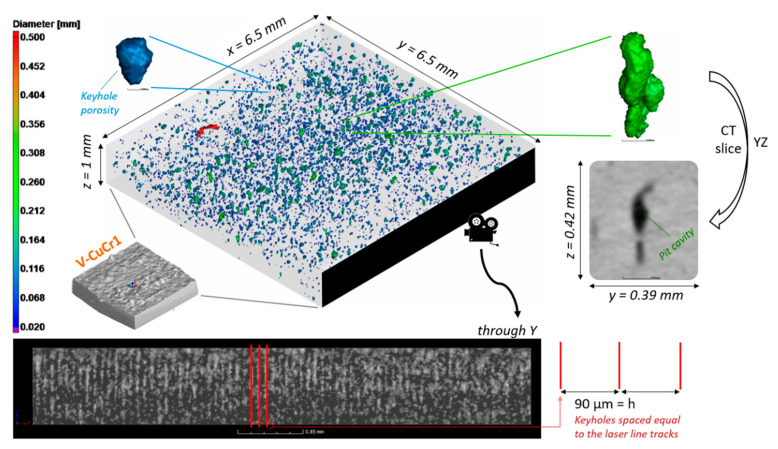
Overview of the porosity distribution in the V-CuCr1 sample, comprising a color-coded 3D view of the porosities, zoomed inserts of two exemplary pores, and a view along the Y direction of the sample to highlight the distribution of the defects following the LPBF hatch spacing of 90 μm.

A first overview of the porosity distribution for the V-CuCr1 is depicted in Figure 5. The total porosity count for the sample was 6269 pores, with a calculated defect volume ratio of 0.66%. No inclusions were detected, neither through visual inspection nor by the identification algorithm.

It becomes clear from the 3D view how most of the detected porosities are below 100 μm in diameter (circumscribed sphere, *Ø_cs_*), with only some sparse pore cavities present that span multiple build layers. Cavities and pits normally are the result of the stacking of multiple pores due to particles ejection and/or keyhole mode of melting, which are both consequences of an excessive energy input [9]. The keyhole mode of melting is a limiting factor of IR reflective materials processed via LPBF, like copper, since their optical absorption changes drastically from solid state to liquid [41]. Hence, while a high *E_v_* is needed to start the melting process, the same high *E_v_* causes vortices, metal vaporization, and high fluid speeds entrapping metal vapors or shielding gases at the bottom of the laser line track. This behavior was discussed already by Jadhav et al. for different copper alloys [17,42] and is one of the causes for their small LPBF processing windows.

**Figure 6:**
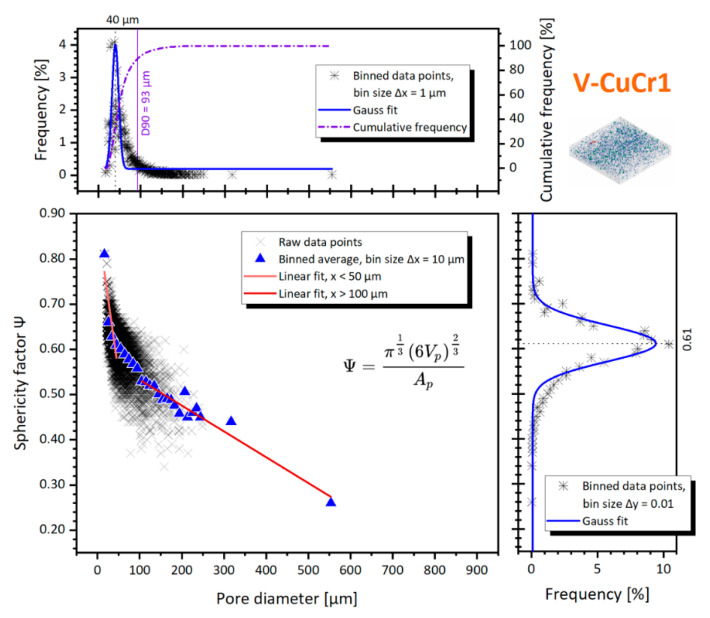
Pore diameter against the sphericity factor for the V-CuCr1 sample; inserts with percentage frequency distributions of both sphericity and diameter are included.

The presence of pit cavities combined with the deep melt pools identified on the etched cross-sections (Figure 3c) are indicators to catalogue the bulk of the pores as mainly keyhole pores. This is also clearly suggested by both the shape and size of the pores and their spatial distribution. The shape was evaluated by the sphericity factor (*ψ*), which is plotted in Figure 6 against the pore diameter.

The CT distribution of the pores is centered around *Ø_cs_* = 40 ± 8 μm, with a distribution span D10–D90 of 21–93 μm. Bigger pores are sporadic, with pit cavities and only one considerable void of *Ø_cs_* = 554 μm. At the same time, the sphericity factor has a D10–D90 distribution span of 0.38–0.66. It must be noted that, due to the micro-CT limits, the identified pores might suffer both from a discretization of their surface due to the CT voxel resolution and the meshing performed by the analysis software. This influences their area calculation and thus the sphericity number, which is on average systematically underestimated with an error of around −30% (see [43], chapter 6). Nevertheless, as stated before, all the factors can directly be compared between the three CuCr1 specimens.

Linear fits of different portions of the data are also included in Figure 6 to ease the comparison through the lines gradients. The linear fits are performed arbitrarily for the 50 > *Ø_cs_* > 100 μm to evidence the behavior of smaller pores vs. bigger pores. Their gradients correspond to −6.65 vs. −0.57, showing how the pores bigger than 100 μm decrease slowly their shape factor at the increase of their diameter, compared to the smaller defects. We want to denote that the gradient of the region *Ø_cs_* > 100 μm is largely influenced by the outlier void at *Ø_cs_* = 554 μm, yet, as shown in Section 3.2.2, without this outlier the difference in the gradient value would be even greater compared to the N2-CuCr1 sample.

The large spread of data points concentrated in the transition zone 50 < *Ø_cs_* < 100 μm is probably due to the expected mixture of small spherical metallurgical pores together with keyhole ones. This is expected, since metallurgical pores are also favored at slow scanning speeds, entrapping more gases within the melt pool or evolved from the powder during consolidation [44]. On the other hand, the non-spherical shape of most of the keyhole porosities is probably a consequence of keyhole instability, a cause of the high thermal conductivity of Cu which promotes the rapid dissipation of heat and therefore induces the collapse of the keyhole cavity quickly after its formation [45]. Collapsing of the keyhole, favored by the non-uniform distribution of the laser energy along the keyhole wall [46], happens because of the loss of balance of the liquid metal under the action of gasified metal recoil pressure, liquid metal surface tension, static liquid pressure, and gravity. This effect, described by Wang et al. for LPBF AlSi10Mg [47] and by Bayat et al. for LPBF Ti6Al4V [48], is more evident for copper as a consequence of the material’s physical properties already discussed in the introduction.

Finally, from the shape analysis perspective, the last important take-away is the projected size of the larger pores in the Z direction (LPBF building direction). As depicted in the lower inserts of Figure 5, elongated pit cavities—like the green pore example—can span different layer thicknesses. On average, the cavities present a projected Z size of 0.07 ± 0.03 mm with a maximum of 0.31 mm. This is a pore extending for more than 10 LPBF building layers. While these kinds of porosities, if limited in number, can be negligible in regards of the mechanical and electrical/thermal properties, they might hinder applications like conformal cooling or general indirect-contact heat exchangers with thin sections [49], where a perfect sealing is deemed critical.

**Figure 7:**
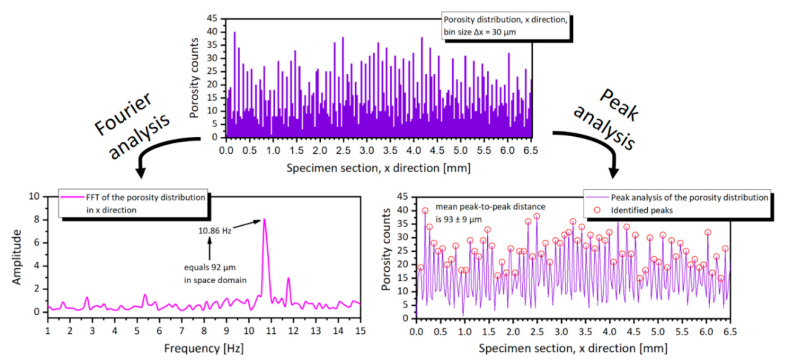
Porosity distribution along the X direction, for the V-CuCr1 sample; a Fourier analysis and a peak analysis is performed on the signal to highlight its fundamental frequency.

The lower insert of Figure 5 is also the starting point to describe the spatial distribution of the defects inside the sample. The qualitative image can be evaluated quantitatively, using the position of the center of mass for each pore in relation to the aligned coordinate system of the CT 3D data. The calculated frequency distribution, for example in the X direction, is depicted in Figure 7 (top).

From the porosity distribution, a Fourier analysis was performed to highlight the fundamental frequencies of the space domain. The signal amplitude vs. frequency plot in Figure 7 (bottom left) presents a main frequency at 10.86 Hz, which coincides with a 92 μm period in the considered space domain. The calculated number corresponds almost perfectly to the employed LPBF hatch spacing (*h*) of 90 μm, further corroborating the hypothesis of an uneven distribution of the porosity due to the alignment of the pores at the bottom of each laser scan track while melting in keyhole mode. The same conclusion can be achieved if a simpler peak analysis [50] is carried out directly in the space domain. In such a way, a mean peak-to-peak distance can be calculated with its standard deviation (Figure 7, bottom right), which amounts to 93 ± 9 μm for the case in question. The standard deviation provides a useful insight on the distribution of the pores around the center line of the scan tracks; this deviation can be used to further compare results of the V-CuCr1 against the other samples.

#### 3.2.2. Micro-CT of the N2-CuCr1 Specimen

**Figure 8:**
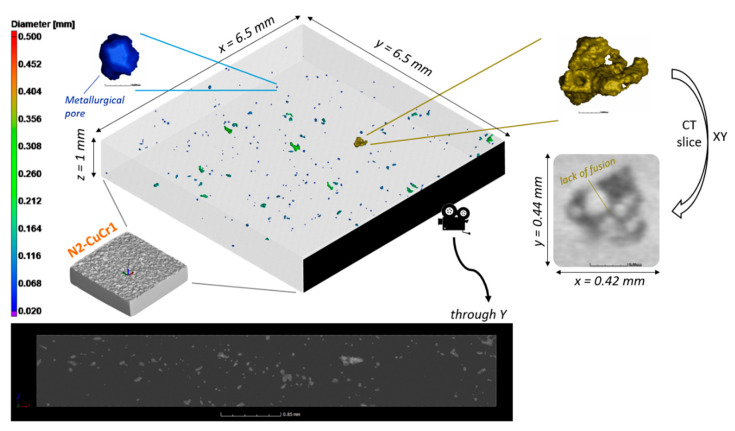
Overview of the porosity distribution for the N2-CuCr1 sample, comprising a color-coded 3D view of the porosities, zoomed representative metallurgical, and lack-of-fusion pores, as well as a 2D view through the Y direction of the sample.

A first overview of the porosity distribution for the N2-CuCr1 is depicted in Figure 8. The total porosity count for the sample was 266 pores, with a calculated defect volume ratio of 0.05%. No inclusions were detected, neither through visual inspection nor the identification algorithm.

The overview in Figure 8 and the calculated defect volume ratio depict a sample having almost full density, in contrast with the Archimedes result of 99.1% relative density. However, we are aware from the discussion at the beginning of Section 3.2 that the CT analysis is known for reporting on average a lower porosity content, and this can be accentuated if most of the pores are small metallurgical pores with a size below the detection threshold of the CT scan. Accordingly, it can be concluded that the N2-CuCr1 specimen processed in optimal conditions, without any contamination, develops very small defects, the majority of which are below the CT threshold of 8 voxel sizes in volume. This is also in line with the analysis of the porosity size distribution for the CN2-CuCr1 specimen, which highlighted the coexistence of small defects together with the larger lack-of-fusion pores (Figure 13, in the following Section 3.2.3). Surprisingly, in N2-CuCr1, few lack-of-fusion pores are detected, too, probably caused by local variations, e.g., a cause of oxidized powder particles, spatters-induced defects, not optimally surface-modified particles, or other unaccounted local combination of effects during the LPBF fabrication.

**Figure 9:**
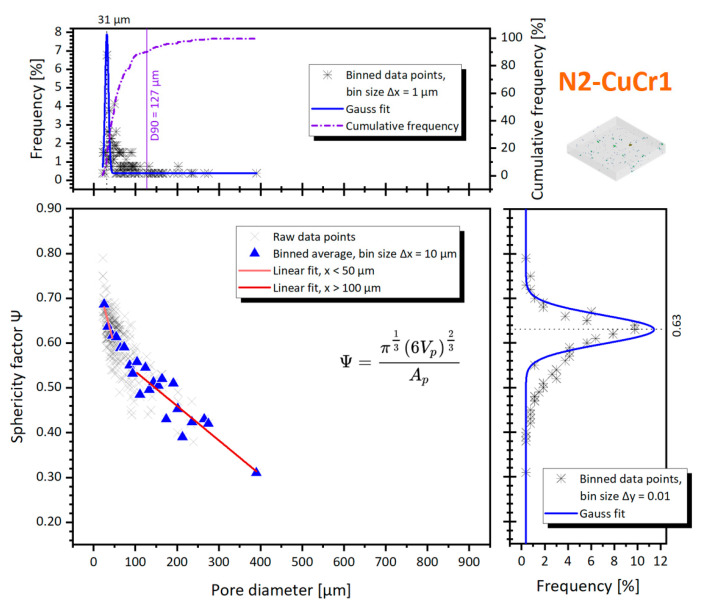
Pore diameter against the sphericity factor, for the N2-CuCr1 sample; inserts with percentage frequency distributions of both sphericity and diameter are included.

For completeness, the sphericity factor is also plotted in Figure 9 for N2-CuCr1, against the pore diameter.

The Gaussian fit of the pore size distribution in Figure 9 confirms that the porosities in N2-CuCr1 are skewed toward smaller defects, centered around *Ø_cs_* = 31 ± 4 μm. The sphericity factor mean fitted value is also slightly higher compared to V-CuCr1, implying that, on average, the detected defects are more spherical. However, with this low amount of porosities’ data points in N2-CuCr1, the frequency distributions are to be taken with a grain of salt, being highly influenced by the bigger pores which are easier to be detected by CT. For the same reason of limited data points, the spatial distribution analysis is not presented.

In the end, it goes without saying that the N2-CuCr1 sample and therefore the surface-modified CuCr1 formulation offers the most favorable evolvement of defects, with the least amount of porosities, a beneficial size and shape of the pores, and no detectable anisotropy in their spatial distribution. Therefore, anisotropies in mechanical, thermal, or electrical properties are limited, and an improvement of the ductility is expected [31] compared to V-CuCr1. Other effects, such as the anisotropy induced by preferential orientations in the crystallographic texture developments [21], are yet to be studied.

#### 3.2.3. Micro-CT of the CN2-CuCr1 Specimen

**Figure 10:**
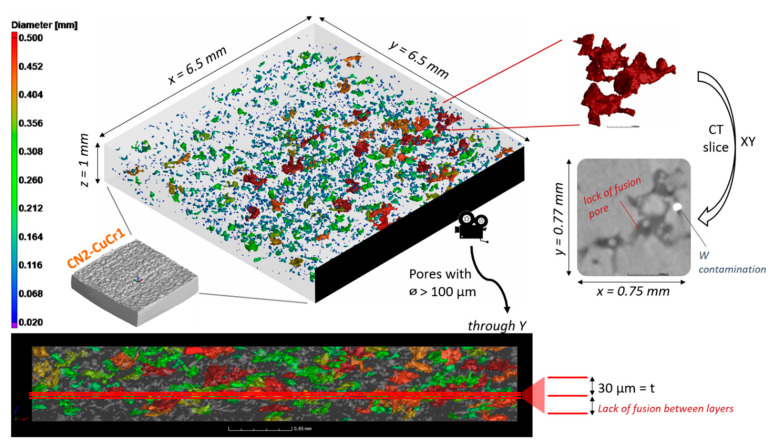
Overview of the porosity distribution for the CN2-CuCr1 sample, comprising a color-coded 3D view of the porosities, a zoomed insert of one exemplary lack-of-fusion pore, and a view through the Y direction of the sample to highlight the distribution of the defects in the building direction Z.

The last specimen in examine consists of a contaminated sample made of surface-modified CuCr1 powder. A first overview of the porosity distribution for the CN2-CuCr1 is depicted in Figure 10. The total porosity count for the sample was 4219 pores, with an initial calculated defect volume ratio of 1.16%.

**Figure 11:**
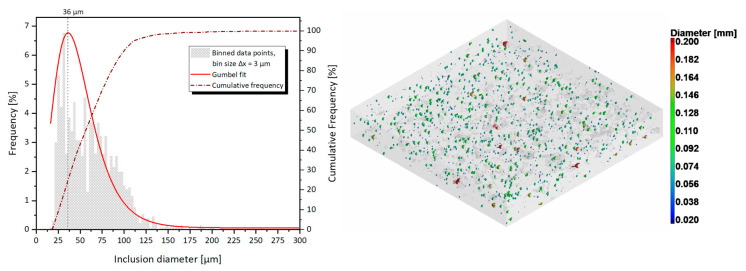
Inclusions size distribution inside the CN2-CuCr1 specimen; the percentage frequency and cumulative frequency are plotted together with a color-coded 3D view (right side).

The overview in Figure 10 confirms the findings discussed in Section 3.1, showing the presence of multiple extended porosities in the XY planes, corresponding to lack of fusion between the LPBF layers. Moreover, an initial guess on the origin of the abnormal porosity count comes from the observation of the 2D CT slices, where bright round spots are encountered throughout the whole analyzed volume. The bright spots, which are related to an unidentified denser material than copper, were therefore quantified through an inclusions identification in the VGSTUDIO software. A total of 1236 inclusions were discovered in the 6.5 × 6.5 × 1 mm^3^ volume, with a 0.14% defect volume ratio. Their percentage frequency and cumulative frequency size distribution are plotted in Figure 11.

The shape and size of the inclusions are compatible with a different LPBF powder, which might have contaminated the CuCr1 build job during the material switch in the LPBF machine. It is also quite straightforward to identify the possible contaminant, tracing back the internal log of users for the AM machine and acknowledging that it must be a material much denser than copper given its strongly deviating gray value in the CT scan. Hence, the inclusions are identified as W (*ρ* = 19.3 g/cm^3^), which was processed right before the contaminated CuCr1 build. Tungsten is a challenging material for LPBF, and normally it is not easily fabricated due to its good thermal conductivity, high melting point, high ductile-to-brittle transition temperature, and high surface tension [51,52]. Furthermore, as copper and tungsten are not mutually soluble and wettable, contaminations of W can effectively hinder the bonding between consecutive LPBF layers of CuCr1.

**Figure 12:**
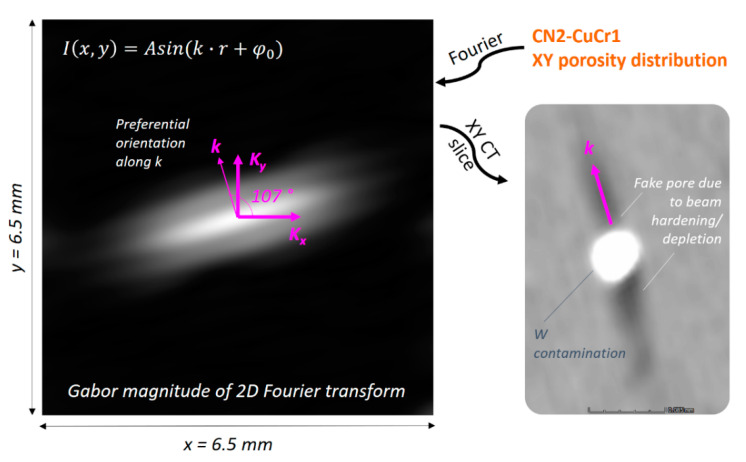
Gabor magnitude of the 2D Fourier transform for the CN2-CuCr1 CT dataset (XY plane), to highlight preferential spatial frequencies; the repeating pattern oriented at 107° is identified as a streaking artefact generated by the W contaminants due to X-ray beam hardening and partial beam depletion.

All duly noted, it remains interesting to compute quantitative numbers of the CN2-CuCr1 porosity to understand how much the W contamination contributed to the defects in comparison to the uncontaminated N2-CuCr1 sample. Nevertheless, to perform the quantification correctly, a differentiation between apparent porosities due to CT artefacts and real porosities was necessary for the sample. The reason behind this differentiation comes from the effect that such a dense contaminant can have on the X-ray beam, resulting in selective attenuation of lower energy photons, i.e., a very localized beam hardening. Beam hardening and partial beam depletion can give rise to visual artefacts in case of heavy contaminants, if the CT settings are only tuned for the material in play (CuCr1) and thus they do not consider the extra X-ray absorption from the heavier inclusion (W). To investigate if the CN2-CuCr1 CT dataset might be affected, a two-dimensional Fourier transform was computed from the 2D CT slices (on the XY plane) to identify preferential spatial frequencies corresponding to repeating patterns in the space domain. Given the very scattered nature of the porosities, a Gabor filter [53] was applied on top of the Fourier transform to visually accentuate the uncovered preferential frequency (Figure 12).

Indeed, the Fourier transform tells that a repeating pattern is present in the space domain and can be traced back to a streaking artefact generated by the tungsten inclusions and oriented at 107° in the XY plane. The artefact is a consequence of X-ray beam hardening and partial beam depletion from the inclusions, and the specific orientation is originated from the inclination of the sample during the CT scan plus the higher absorption of X-rays from the thicker sample section while rotating on the CT manipulator.

**Figure 13:**
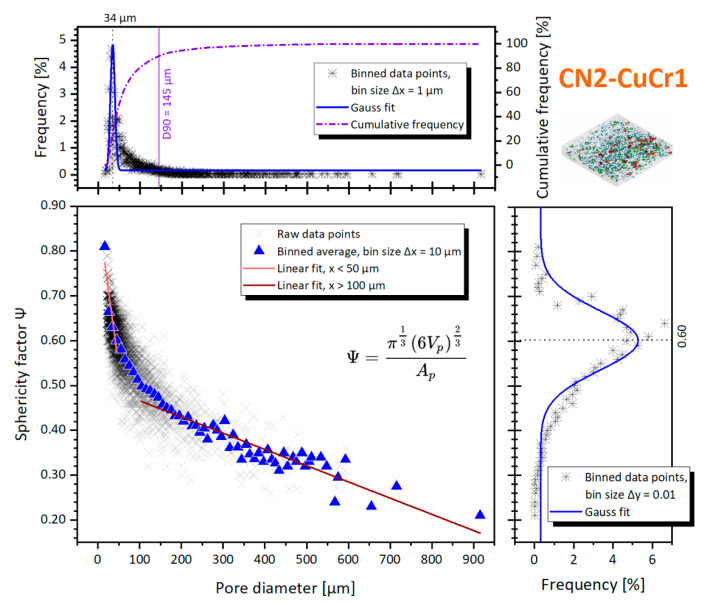
Pore diameter against the sphericity factor for the CN2-CuCr1 sample; inserts with percentage frequency distributions of both sphericity and diameter are included.

Henceforth, a fraction of the initial porosity count for the specimen is instead uncovered as an artefact caused by the nature of the CT scan itself and the unexpected presence of the W contaminant. However, thanks to the performed analysis, these pores can be easily filtered out using their position, shape, and orientation as categorizers. The recalculated porosity count for the sample was 3759 pores (i.e., 460 defects scrapped as artefacts), with a defect volume ratio of 1.11%. Using the recalculated list of pores, the sphericity factor is plotted in Figure 13, against the pore diameter.

The CT distribution of the pores is centered around *Ø_cs_* = 34 ± 6 μm, with a distribution span D10–D90 of 26–145 μm. At the same time, the sphericity factor has a D10–D90 distribution span of 0.32–0.67. It becomes very clear how the CN2-CuCr1 sample presents both a considerably higher number of pores with *Ø_cs_* > 100 μm as well as broader sphericity values, compared to the V-CuCr1 and N2-CuCr1 specimens. This is obviously related to the extensive and irregular lack-of-fusion porosities. However, interestingly, the Gaussian fit of the pore diameters is centered around a lower value than V-CuCr1, suggesting that the CN2 sample has a coexistence of small metallurgical pores together with the larger lack-of-fusion defects. This is in line with what was discussed in Section 3.1 and Section 3.2.2, as the LPBF processing parameters used are leading to conduction mode of melting instead of keyhole.

The linear fit gradients correspond to −6.9 for *Ø_cs_* < 50 μm vs. −0.36 for *Ø_cs_* > 100 μm, showing again how the pores bigger than 100 μm slowly decrease their shape factor at the increase of their diameter, compared to the smaller defects. If the behavior is compared to the V-CuCr1 specimen, the gradient of the small porosities tends to move more quickly to higher sphericities for the absence of non-spherical keyhole pores, while the gradient for bigger porosities moves slower toward low sphericities caused by the broader range of irregular lack-of-fusion pores (the biggest at *Ø_cs_* = 916 μm).

**Figure 14:**
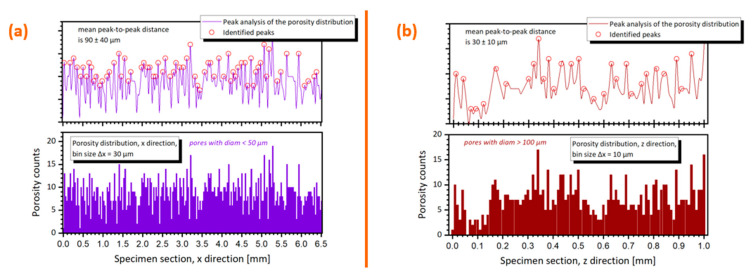
(**a**) Peak analysis porosity distribution along the X direction for the CN2-CuCr1 sample, considering only porosities with *Ø_cs_* < 50 μm; (**b**) Peak analysis porosity distribution along the Z direction for the same sample, considering only porosities with *Ø_cs_* > 100 μm.

Finally, on top of the shape and size analysis, a Fourier and/or peak analysis can be performed to study the spatial distribution of the defects. The peak analysis for the CN2-CuCr1 sample is reported in the Figure 14 for the X and Z directions. The X direction peak analysis considers only the *Ø_cs_* < 50 μm to study how the small pores are allocated in the hatching direction; the Z direction peak analysis considers only the *Ø_cs_* > 100 μm to confirm that bigger pores are mainly distributed in between consecutive layers.

In Figure 14a, a preferential distribution of the small porosities is unraveled, coinciding with the employed LPBF hatch spacing of 90 μm, similar to what was observed for the V-CuCr1 sample. However, the standard deviation of ±40 μm is one order of magnitude larger than the one measured for the peak analysis of V-CuCr1 (Figure 7), implying that the small pores are more equi-distributed in the XY plane, thus less heavily concentrated between repeating *h*. At the same time, in Figure 14b, the average Z spacing of the bigger porosities is equal to *t* ± 10 μm. The considerable standard deviation is both a consequence of the fewer data points available for the calculation (i.e., fewer big pores compared to the small ones) as well as the fact that lack-of-fusion defects often span more than one layer, following the curved boundaries at the bottom of the melt pool (Figure 4b). Similar conclusions are derived with a Fourier analysis, with 90 μm for X direction and 30 μm for Z, corresponding to 11.02 and 32.67 Hz in the frequency domain, albeit those signals are almost buried in the background noise (i.e., they have a low amplitude) confirming the high standard deviations of the peak analysis.

## 4. Summary and Conclusions

The most important descriptors of the porosity analyses performed on the three CuCr1 specimens are summarized in the following Table 1.

With the help of an in-depth micro-CT investigation, this paper has unveiled the different defects evolvement inside high-density specimens produced with virgin CuCr1 powder (V-CuCr1) and a novel [16] surface-modified CuCr1 powder (N2-CuCr1) via the laser powder-bed fusion process. Moreover, a third surface-modified sample was studied (CN2-CuCr1) to uncover the reason behind its abnormal porosity content compared to the N2-CuCr1 sample. In a nutshell, the main takeaways of the research can be summarized in three distinct points:

- It was demonstrated how the use of surface-modified CuCr1 powders for LPBF not only is beneficial in terms of broadening of the CuCr1 LPBF processing window but also for being able to completely eradicate the presence of keyhole-induced porosities thanks to the possibility of achieving dense parts at a considerably lower volumetric laser energy density. In that regard, it was shown that, while a 98.64% dense specimen can be attained from virgin CuCr1 powder with an *E_v_* of 926 J/mm^3^ (V-CuCr1), the same specimen develops both keyhole pores, aligned at the bottom of the laser line track, and pit cavities defects. These pores, being preferentially distributed and—more specifically for the pit cavities—elongated in the building direction for multiple layer thicknesses, can become critical points of failure for applications such as conformal cooling channels or heat exchangers with thin sections where a perfect sealing is deemed critical. Moreover, they can promote anisotropies at the level of the mechanical and thermal/electrical properties of the parts. This behavior can be avoided using CuCr1 surface-modified powders processed at an *E_v_* of 231 J/mm^3^, which led to specimens (N2-CuCr1) having densities >99% and presenting only small, spherical, and equi-distributed metallurgical pores. Therefore, thanks to the micro-CT porosity analysis, the choice between virgin and surface-modified CuCr1 can now be calibrated not only on, e.g., the powder cost or the different achievable LPBF productivity but also on possible limitations coming from undesired defects evolvement in the part.
- It was displayed how the use of micro-CT can aid the optimization of novel LPBF materials, both by providing detailed information on the defect size, shape, and spatial distribution inside a studied specimen as well as possibly uncovering faults such as undesired contamination by a different LPBF powder. In that regard, a 0.14 vol% W content was identified in the CN2-CuCr1 sample, where lack-of-fusion pores were promoted by the contaminant due to the W particles being insoluble with Cu. The 0.14 vol% W content was directly connected to the ~1% decrease in *ρ_rel_* experienced by CN2-CuCr1 compared to N2-CuCr1.
- The main type of porosities which can be found in high-copper-containing alloys were exposed and discussed for the three CuCr1 specimens examined. The LPBF melting mode (keyhole or conductive), the properties of the material, and the potential presence of contaminants were connected to the different types of porosity, their size, and morphology. Moreover, the use of a Fourier transform or a peak-to-peak analysis on the spatial frequency distribution of the pores provided a possible and quick way to derive quantitative information about the defect spatial distribution of defects. This latter method not only confirmed and quantified the preferential distribution of the keyhole and the lack-of-fusion pores, respectively, spaced as the employed LPBF *h* and *t* parameters, but additionally it was critical to separate real defects from CT artefacts in the CN2-CuCr1 sample, which presented fake pores induced by beam hardening/depletion from the W contaminant.

The performed research solely focused on the CT investigation, with the broader goal of promoting the use of metrological CT equipment for the thorough understanding of novel and complex materials manufactured via processing techniques such as LPBF. Combining the information of CT results with the standard mechanical, chemical, physical, and metallurgical characterizations will help to progress further toward a fundamental comprehension of the physics of the LPBF process and the properties of LPBF-fabricated materials.

## 5. Patents

The authors S.D.J. and K.V. declare that a patent cooperation treaty (PCT) application has been filled on the surface-modified copper powder and the method of production with the following details:

Title: Copper, gold, or silver powder for powder-bed additive manufacturing and method of manufacturing such powder.
Patent number: WO2020/099662 A1 (Publication date: 22 May 2020).
First filing date: 15 November 2018.

## Figures and Tables

**Table 1:** Summary of the Archimedes and micro-CT porosity investigations of the three CuCr1 specimens, with relevant descriptors.

Specimen	Archimedes *ρ_rel_*	Micro-CT *ρ_rel_*	Porosity Avg *Ø_cs_* ^1^	Pores’ *Ø_cs_* Span, D10–D90 ^2^	Pores’ *ψ* Span, D10–D90 ^2^	Main Type of Porosity
V-CuCr1	98.64%	99.34%	40 ± 8 μm	21–93 μm	0.38–0.66	Keyholes, pit cavities, metallurgical
N2-CuCr1	99.10%	99.95%	31 ± 4 μm	30–127 ^3^ μm	0.39–0.67	Metallurgical
CN2-CuCr1	98.23%	98.89%	34 ± 6 μm	26–145 μm	0.32–0.67	Lack-of-fusion, metallurgical

^1^ The average circumscribed sphere (Øcs) diameter of the pores is calculated from the center of the Gaussian fit applied on the frequency distribution of the defects from the CT analysis. ^2^ D90 signifies that 90% of the defects have Øcs/ψ below this value; hence, D10–D90 is used to describe the span of Øcs/ψ of the pores, measured from the CT analysis. ^3^ The Øcs span of N2-CuCr1 is heavily influenced by the low amount of porosities detected.

## Data Availability

The datasets generated during and/or analyzed during the current study are available from the corresponding authors on reasonable request.
